# Expression of the ubiquitin-proteasome pathway and muscle loss in experimental cancer cachexia

**DOI:** 10.1038/sj.bjc.6602780

**Published:** 2005-09-06

**Authors:** J Khal, S M Wyke, S T Russell, A V Hine, M J Tisdale

**Affiliations:** 1 Pharmaceutical Sciences Research Institute, Aston University, Birmingham B4 7ET, UK

**Keywords:** cachexia, protein degradation, ubiquitin-proteasome proteolysis, gastrocnemius muscle

## Abstract

Muscle protein degradation is thought to play a major role in muscle atrophy in cancer cachexia. To investigate the importance of the ubiquitin-proteasome pathway, which has been suggested to be the main degradative pathway mediating progressive protein loss in cachexia, the expression of mRNA for proteasome subunits C2 and C5 as well as the ubiquitin-conjugating enzyme, E2_14k_, has been determined in gastrocnemius and pectoral muscles of mice bearing the MAC16 adenocarcinoma, using competitive quantitative reverse transcriptase polymerase chain reaction. Protein levels of proteasome subunits and E2_14k_ were determined by immunoblotting, to ensure changes in mRNA were reflected in changes in protein expression. Muscle weights correlated linearly with weight loss during the course of the study. There was a good correlation between expression of C2 and E2_14k_ mRNA and protein levels in gastrocnemius muscle with increases of 6–8-fold for C2 and two-fold for E2_14k_ between 12 and 20% weight loss, followed by a decrease in expression at weight losses of 25–27%, although loss of muscle protein continued. In contrast, expression of C5 mRNA only increased two-fold and was elevated similarly at all weight losses between 7.5 and 27%. Both proteasome functional activity, and proteasome-specific tyrosine release as a measure of total protein degradation was also maximal at 18–20% weight loss and decreased at higher weight loss. Proteasome expression in pectoral muscle followed a different pattern with increases in C2 and C5 and E2_14k_ mRNA only being seen at weight losses above 17%, although muscle loss increased progressively with increasing weight loss. These results suggest that activation of the ubiquitin-proteasome pathway plays a major role in protein loss in gastrocnemius muscle, up to 20% weight loss, but that other factors such as depression in protein synthesis may play a more important role at higher weight loss.

Loss of skeletal muscle is a prominent feature of cancer cachexia, resulting in weakness, immobility and finally death. Body composition analysis of lung cancer patients who had lost 30% of their body weight revealed a 75% reduction in skeletal muscle mass, while visceral proteins were preserved (Fearon, 1992). Loss of skeletal muscle protein is thought to arise from a suppression of protein synthesis (Emery *et al*, 1984) and/or an increase in protein breakdown and oxidation of amino acids (O'Keefe *et al*, 1990).

A number of experimental animal tumours have been developed to study the mechanism of muscle wasting in cachexia. In the murine MAC16 colon adenocarcinoma model, weight loss occurs with small tumour burden (less than 0.1% of host weight), and without a reduction of food and water intake (Beck and Tisdale, 1987), allowing a study of the metabolic components of cachexia. As in cancer patients, the major components of body weight loss in the MAC16 model are adipose tissue and skeletal muscle mass. Loss of skeletal muscle mass arises from a reduction (by 60%) in protein synthesis and an increase (three-fold) in protein degradation (Beck *et al*, 1991). As was found in a rat model of cancer cachexia (Baracos *et al*, 1995; Llovera *et al*, 1997), the major contribution to the loss of muscle mass in mice bearing the MAC16 tumour appears to arise from an upregulation of the ATP-ubiquitin-dependent proteolytic pathway (Lorite *et al*, 1998), as reflected by increased levels of ubiquitin-conjugated proteins and increased mRNA levels for the 14 kDa ubiquitin carrier protein, E2 and the C9 proteasome subunit in gastrocnemius muscle. However, as with other studies in animals this was only measured after weight loss had developed, and there have been no measurements of the expression of the major components of the ubiquitin-proteasome pathway at different extents of weight loss, or a comparison between different muscle types to understand if this pathway totally explains protein degradation. However, a study in gastric cancer patients showed an increase in expression of ubiquitin mRNA and proteasome activity compared with controls, and proteasome activity correlated both with stage of disease and weight loss (Bossola *et al*, 2003).

The purpose of the present study was to measure changes in expression of the key regulatory components of the ubiquitin-proteasome proteolytic pathway in skeletal muscle with the development of cachexia in the MAC16 model. Protein degradation in this model has been shown to increase progressively with increasing weight loss between 15 and 30%, up to a maximum increase of 240% at a weight loss of 30% (Smith and Tisdale, 1993a). Expression of mRNA for E2, and the proteasome subunits C2 and C5, representing *α* and *β* subunits of the proteasome respectively, have been determined in gastrocnemius and pectoral muscle by quantitative RT–PCR as a measure of the ubiquitin-proteasome pathway. These are considered to be representative of proteasome structural and catalytic subunits indicative of proteasome expression. Previous studies (Lorite *et al*, 2001; Whitehouse *et al*, 2001) have shown that subunits of the 19S regulator change in concert with the proteasome *α*-subunit. As a measure of ubiquitin conjugation mRNA levels for E2_14k_ have been determined, which was originally suggested to be rate-limiting for ubiquitin conjugation during starvation (Wing and Banville, 1994), although studies in E2_14k_ knockout mice suggest that it is nonessential (Adegoke *et al*, 2002). However, recent data suggest that the ubiquitin-protein ligases (E3), muscle ring-finger 1 (MURF1); (Bodine *et al*, 2001) and muscle atrophy F box (MAFbx)/atrogin-1 (Gomes *et al*, 2001), may be the rate-limiting step in various conditions of muscle atrophy including cachexia.

In this study, the expression of some components of the ubiquitin-proteasome pathway has been monitored with increasing weight loss in gastrocnemius and pectoral muscle of mice bearing the MAC16 tumour, since some reports (Khal *et al*, 2005) suggest that the expression of this pathway in the skeletal muscle of cancer patients decreases at weight losses greater than 20%. Measurements of protein levels of proteasome *α*-subunits and E2_14k_ have also been determined by immunoblotting to ensure that changes in mRNA are reflected in changes in protein expression.

## MATERIALS AND METHODS

### Materials

Mouse monoclonal antibody to 20S proteasome subunits *α*1, 2, 3, 5 and 7 (clone MCP231) was purchased from Affinity Research Products (Exeter, UK). Rabbit polyclonal antisera to E2_14k_ was a gift from Dr Simon Wing (McGill University, Montreal, Canada). The antibody detected E2_14k_ as a *M*_r_ 17 kDa band (Rajapurohitam *et al*, 1999). Goat polyclonal antiserum to MURF3 (E3) and peroxidase-conjugated rabbit polyclonal antisera to goat IgG were purchased from Abcam Ltd (Cambridge, UK). Mouse monoclonal anti-PIF antibody was prepared as previously (Todorov *et al*, 1996). Peroxidase-conjugated goat anti-rabbit and rabbit anti-mouse secondary antibodies were from Dako Ltd (Cambridge, UK).

Wizard™ mini or maxi preps, used to prepare plasmid DNA from overnight cultures of bacterial clones, were obtained from Promega (Southampton, UK), as were the T7 RNA polymerase kit, RNAsin inhibitor, reverse transcriptase, reverse transcription buffer and PCR grade magnesium chloride. *Taq* polymerase and Hybond C were from Amersham Biosciences UK Ltd (Bucks, UK), TRI reagent for RNA isolation was purchased from Sigma-Aldrich Co-Ltd (Dorset, UK), while the oligonucleotide primers were from MWG Biotech (Ebersberg, Germany). RNA storage buffer was purchased from Ambion Ltd (Cambridgeshire, UK) and PCR buffer was from Roche, Switzerland.

### Animals

All animal experiments have been carried out with ethical committee approval. The ethical guidelines that were followed met the standards required by the UKCCR guidelines (Workman *et al*, 1998).

Pure strain NMRI mice were obtained from our own inbred colony and were fed a rat and mouse breeding diet (Special Diet Services, Witham, UK). Fragments of the MAC16 colon adenocarcinoma, excised from donor animals with established weight loss, were implanted into the flanks of NMRI mice by means of a trocar, as described (Beck and Tisdale, 1987). Animals started to lose weight approximately 10–12 days after transplantation. Animals were terminated at various extents of weight loss, and both gastrocnemius and pectoral muscles were removed, and immediately frozen in liquid nitrogen, and stored at −70°C before further analysis.

### Measurement of protein degradation *ex vivo*

Gastrocnemius muscles were excised from mice bearing the MAC16 tumour, and with varying extents of weight loss, and preincubated for 45 min in 3 ml oxygenated (95% oxygen/5% carbon dioxide) Krebs–Henseleit bicarbonate buffer, pH 7.4, containing 5 mmol^−1^ glucose and 0.5 mmol l^−1^ cycloheximide. Protein degradation was determined by the release of tyrosine (Waalkes and Udenfriend, 1957) over a 2 h period in the absence and presence of lactacystin (10 *μ*M). Tyrosine release, which was blocked in the presence of lactacystin, was considered to be proteasome specific.

### Measurement of proteasome activity

The ‘chymotrypsin-like’ enzyme activity of the proteasome was measured fluorometrically as described by Orino *et al* (1991). Muscles were removed from animals with varying degrees of weight loss and homogenized with a teflon glass homogenizer in 20 mM Tris-HCl, pH 7.5, 2 mM ATP, 5 mM MgCl_2_ and 1 mM DTT on ice at 4°C. The homogenate was further dissociated by sonication at 4°C, and the sonicate was then centrifuged at 18 000 **g** for 10 min, and the supernatant was used to measure enzyme activity by the release of aminomethylcoumarin (AMC) from succinyl-LLVY-AMC. The reaction was performed for 1 h at room temperature in 100 mM Tris. HCl, pH 8.0, and was terminated by the addition of 80 mM sodium acetate, pH 4.3. The fluorescence of AMC was determined with an excitation wavelength of 360 nm and an emission wavelength of 460 nm. The reaction was performed in the presence and absence of the specific proteasome inhibitor lactacystin, and only lactacystin suppressible activity was considered to be proteasome specific. The activity was adjusted for the protein concentration of the sample, determined using the Bradford assay (Sigma Aldridge, Dorset, UK).

### Quantitative RT–PCR

Total RNA was extracted from muscle using TRI-reagent and the RNA concentration was determined from the absorbance at 260 nm. To quantitate the mRNA of interest, increasing amounts of competitor RNA, which differed from the mRNA of interest by containing a short deletion, were added to 250 ng of total RNA and coamplified using RT–PCR. The amount of specific mRNA was calculated from the amount of competitor, when equal amounts of PCR product were obtained from the competitor and target RNA.

The C2 proteasome subunit mRNA was amplified using the primer pairs: 5′-CGCACGGAGTGCTGGTTGCAC-3′ (forward) and 5′-GTACGAGCTGATTGAGAACGG-3′ (reverse) and corresponds to positions 176–187 and 531–552 bp. The competitor primer was designed to produce a DNA fragment containing a 76-bp deletion by producing a loop in the DNA during the PCR amplification process. The sequence of this primer was 5′-GTACGAGCTGATTGAGAACGG**CATAACCAGCAATGAGCAGCC**-3′, which corresponds to the reverse complement of bases 561–552 and **435–455** of the mouse C2 gene. The competitor product was 309 bp. The C5 proteasome mRNA was obtained from the mouse gene using the primer pairs: 5′-TCAACGGAGGTACTGTATTGG-3′ and 5′-GCATGGCACTTGCTGAGCC-3′ at positions 101–121 and 496–514 bp. The competitor primer was designed 117 bp upstream of the reverse primer producing a product 277 bp long. The sequence of this primer was 5′-GCATGGCACTTGCTGAGC**CGCATGGCACTTGCTGAGCC-**3′, which corresponds to the reverse complement of bases 496–514 and **358–377** of the mouse C5 gene. The E2_14k_ competitor was obtained from the rat gene using the primer pairs: 5′-CTCATGCGGGATTTCAAGCG-3′ and 5′-CTCTTCTCATACTCCCGTTTGCAT-3′. The competitor primer contained a 111 bp deletion and the sequence was 5′-CTCTTCTCATACTCCCGTTGCAT**CGCTTCTGCAGGATGTC**, which corresponds to the reverse complement of bases 443–468 and **312–321** of the rat gene. The competitor DNAs were blunt ended and then ligated into a pET30a vector, which had been blunt-end cut with *Sma*I restriction enzyme. The ligated vector was used to transform DH5*α* competent cells. Plasmid DNA was prepared from PCR positive clones using Wizard-mini-prep. Competitor RNA was produced using T7 RNA polymerase kit, and quantitated using the optical density at 260 nm. Six serial two-fold dilutions were prepared containing known concentrations of competitor. The particular dilutions used were selected to span the selected concentration of C2, C5 and E2_14k_ in the sample.

To synthesise the cDNA template, a mixture consisting of 250 ng target RNA, the particular dilution of the competitor RNA and 0.5 *μ*g of random hexamer was incubated at 70°C for 5 min in a thermal cycler (Genetic Research, Instrumentation Ltd, Essex, UK) and then chilled on ice before the addition of 2.5 *μ*l 5 × reverse transcription buffer, 3 *μ*l of 10 mM each of dATP, dGTP, dCTP, dTTP, 5 units of RNAsin inhibitor and 1 unit reverse transcriptase in a total volume of 12.5 *μ*l. Incubation was at 37°C for 1 h. For the amplification of the cDNA by PCR, 50 *μ*l of PCR mix was added to each tube. The PCR mixture contained 1 × PCR buffer (without magnesium) together with 3 mM MgCl_2_ (E2), 3.5 mM MgCl_2_ (C5) and 2.5 mM MgCl_2_ (C2) together with 1 U *Taq* polymerase. For the E2 gene 10 pmol of each of the primers 5′-CTCATGCGGGATTTCAAGCG-3′ and 5′-CTCTTCTCATACTCCCGTTTG-3′ were used, while for the C5 gene 10 pmol of each of the primers 5′-TCAACGGAGGTACTGTATTGG-3′ and 5′-GCATGGCACTTGCTGAGCC-3′ were used. For the C2 gene, 20 pmol of each of the primers 5′-CGCACGCAGTGCTGGTTGCAC-3′ and 5′-GTACGAGCTGATTGAGAACGG-3′ were used. The temperature-cycling profile for amplification was as follows: 95°C for 2 min for one cycle followed by 95°C for 30 s, 58°C annealing for 1 min and 72°C extension for 1 min for 30 cycles. Control reactions containing all components except reverse transcriptase and another without template were carried out alongside each experiment to show that the RNA (both target and competitor) had no DNA contamination, while the second control showed that there was no contamination in the PCR mixture. Coamplification of E2 target and competitor produced DNA fragments of 395 and 284-bp, respectively; C2 target and competitor produced 385 and 309-bp fragments and C5 target and competitor produced 414 and 297-bp fragments, respectively.

For analysis of results, 15 *μ*l of PCR products were separated on a 2% (w v^−1^) agarose gel containing ethidium bromide. The gel images were visualised on a UV transilluminator and photographed. The intensity of the bands was quantitated using a Phoretix photo-imager programme. The volumes of the competitor and target bands were plotted against the known serial dilutions of the competitor used in the experiment. The amount of the sample RNA corresponds to the amount of competitor when the ratio of competitor to target is 1.0.

### Western blot analysis

Cytosolic protein (5 *μ*g for E2 and 20S proteasome *α*-subunits) was resolved on 10% sodium dodecylsulphate, polyacrylamide gels. Proteins were then transferred to nitrocellulose membranes (Hybond C), which had been blocked with 5% Marvel in Tris buffered saline (TBS) at 4°C overnight. The primary and secondary antibodies, peroxidase conjugated either rabbit anti-goat, goat anti-rabbit or rabbit anti-mouse IgG, were used at a 1 : 1500 dilution. Incubation was for 1 h at room temperature and development was by enhanced chemiluminescence (ECL) (Amersham). Blots were scanned by a densitometer to quantitate differences, and a parallel gel was stained by coomassie blue stain to ensure equal loading. The intensity of bands was quantitated using ‘Phoretix 1D Advanced’ software as above.

### Statistical analysis

Results were expressed as means±s.e.m. of three separate determinations on different animals. Differences were determined by one-way analysis of variance (ANOVA) followed by Tukey–Kramer multiple comparison test. *P*-values less than 0.05 were considered to be significant.

## RESULTS

**Figure 1:**
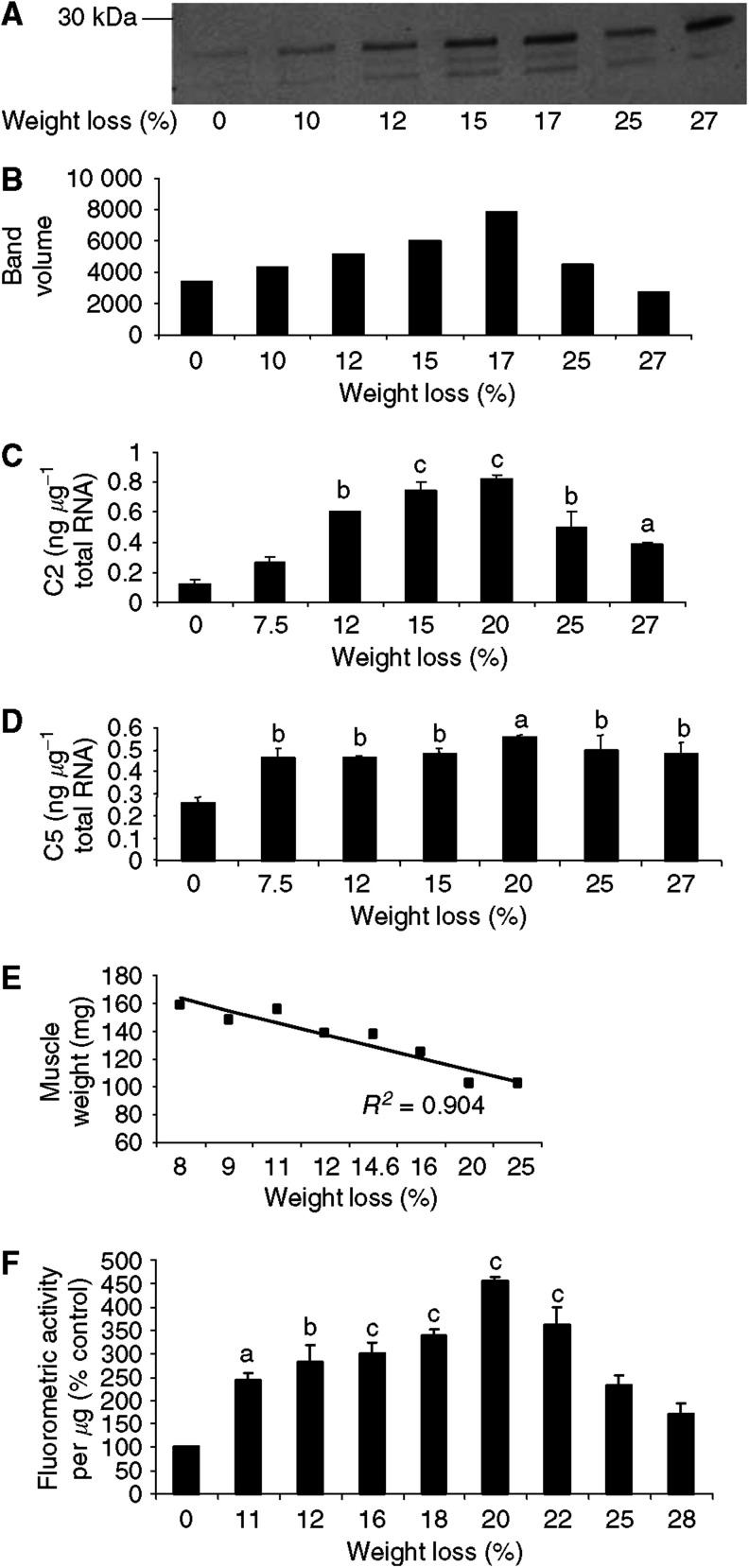
(**A**) Expression of 20S proteasome *α*-subunits in soluble fractions of gastrocnemius muscles of mice bearing the MAC16 tumour and with different extents of weight loss, as detected by immunoblotting. (**B**) Densitometric analysis of band intensities in (**A**) reported in arbitary units. (**C**) Expression of C2 mRNA and (**D**) expression of C5 mRNA in gastrocnemius muscle from mice with different extents of weight loss. Differences from animals without weight loss are expressed as a, *P*<0.05, b, *P*<0.01 and c, < 0.001. (**E**) Relationship between weight of gastrocnemius muscle and percentage weight loss. (**F**) Proteasome proteolytic activity, as measured by the ‘chymotrypsin-like’ enzyme activity, in gastrocnemius muscle of mice with different extents of weight loss. The symbols for significance are the same as in (**D**).

The effect of increasing weight loss on expression of C2 and C5 mRNA as well as 20S proteasome *α*-subunit protein levels, detected by Western blotting, and gastrocnemius muscle weights of mice bearing the MAC16 tumour is shown in Figure 1. Although the monoclonal antibody to the 20S proteasome reacts with six different *α*-type subunits, only three bands were apparent at approximate *M*_r_ 29, 32 and 35 kDa (Whitehouse *et al*, 2001) (Figure 1A). There was a good correlation between expression of C2 mRNA (Figure 1C) and proteasome protein (Figure 1B), with a 6–8-fold increase in C2 mRNA with increasing weight loss from 12%, reaching a maximum at 15–20% weight loss. Although C2 mRNA expression at 25–27% weight loss was significantly enhanced above that found in animals without weight loss (Figure 1C), it was significantly decreased (*P*<0.05) compared with animals exhibiting 20% weight loss. In contrast, expression of C5 mRNA was significantly increased (by two-fold) at all weight losses (Figure 1D), with no significant difference between animals with 7.5% weight loss and those with 27% weight loss. Gastrocnemius muscle weight was directly proportional to total body weight loss, showing progressive loss of skeletal muscle as the cachexia progresses (Figure 1E). Proteasome functional activity in gastrocnemius muscle as determined by the ‘chymotrypsin-like’ enzyme activity, the dominant catalytic activity of the *β*-subunits of the proteasome, was found to follow a similar change with weight loss as expression of protein and mRNA of proteasome *α*-subunits, with an initial increase with weight loss up to 20% followed by a decrease at higher weight loss (Figure 1F).

**Figure 2:**
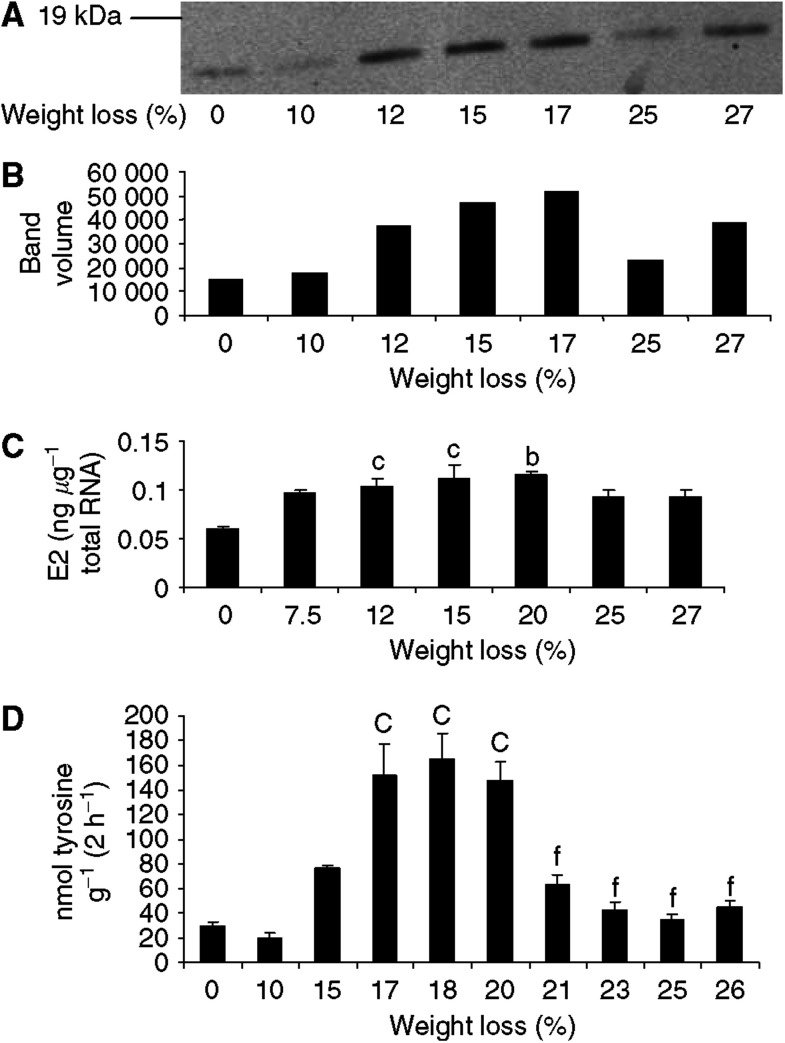
(**A**) Expression of E2_14k_ in soluble extracts of gastrocnemius muscles from mice bearing the MAC16 tumour and with different extents of weight loss, as detected by immunoblotting. (**B**) Densitometric analysis of band intensities in (**A**) reported in arbitary units. (**C**) Expression of E2 mRNA in gastrocnemius muscle from mice with different extends of weight loss. (**D**) Total protein degradation, as measured by tyrosine release, in gastrocnemius muscle of mice with different extents of weight loss. The figures represent protein degradation inhibited by lactacystin (10 *μ*M). Differences from animals without weight loss are expressed as b, *P*<0.01 or c, *P*< 0.001.

The effect of progressive weight loss on the expression of the ubiquitin conjugating protein E2 in gastrocnemius muscle followed a similar pattern to that observed with proteasome *α*-subunits (Figure 2). Thus, expression of E2 mRNA was increased two-fold in gastrocnemius muscle of mice with 12% weight loss and remained elevated up to 20% weight loss (Figure 2C). The expression of E2 mRNA in gastrocnemius muscle of mice with 25 and 27% weight loss was not significantly different from that in mice without weight loss (Figure 2C). The expression of E2_14k_ protein, detected by Western blotting, followed a similar pattern reaching maximal expression at 15–17% weight loss (Figure 2A, B). Proteasome-specific protein degradation in gastrocnemius muscle, determined by the release of tyrosine in the presence of the specific proteasome inhibitor lactacystin (Fenteany and Schreiber, 1998), also peaked between 17 and 20% weight loss and then decreased at higher weight loss (Figure 2D). These results suggest that the ubiquitin-proteasome proteolytic pathway plays a minor role in the degradation of proteins in gastrocnemius muscle at weight loss greater than 20%.

**Figure 3:**
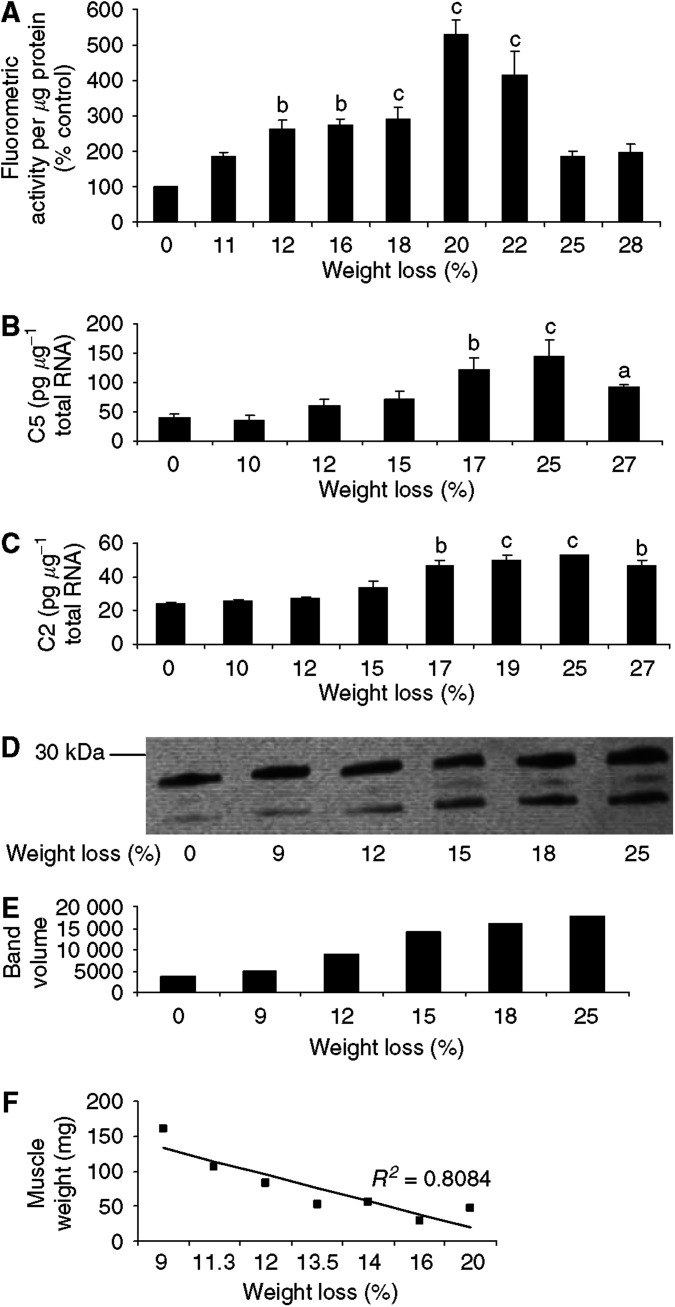
(**A**) Proteasome proteolytic activity, as measured by the ‘chymotrypsin-like’ enzyme activity, in pectoral muscle of mice bearing the MAC16 tumour and with different extents of weight loss. (**B**) Expression of C5 mRNA and (**C**) expression of C2 mRNA in pectoral muscle from cachectic mice bearing the MAC16 tumour. (**D**) Expression of 20S proteasome *α*-subunits, determined by immunoblotting, in soluble extracts of pectoral muscle from mice bearing the MAC16 tumour and different extents of weight loss. (**E**) Densitometric analysis of band intensities in (**D**). Differences from animals without weight loss are expressed as a, *P*<0.05, b, *P*<0.01 and c <0.001. (**F**) Relationship between weight of pectoral muscle and percentage weight loss.

**Figure 4:**
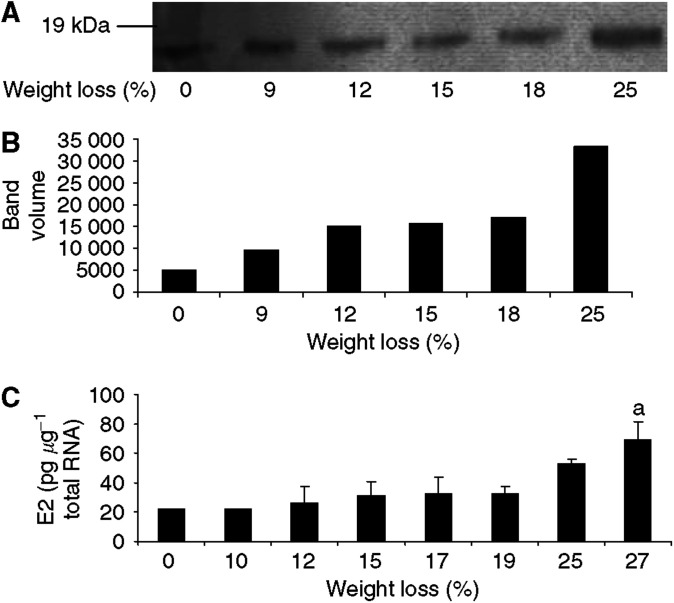
(**A**) Expression of E2_14k_ in soluble extracts of pectoral muscle from mice bearing the MAC16 tumour and different extents of weight loss, detected by immunoblotting. (**B**) Densitometric analysis of band intensities in (**A**) reported in arbitary units. (**C**) Expression of E2 mRNA in pectoral muscle of mice with different extents of mice loss. Differences from animals without weight loss are expressed as a, *P*< 0.05.

Proteasome functional activity in pectoral muscle followed a similar pattern to that found in gastrocnemius muscle (Figure 3A) peaking at weight loss between 18 and 22%. Expression of mRNA for the catalytic *β* subunit (C5) also peaked at weight loss between 17 and 25% with a decrease at higher weight loss (Figure 3B). However, expression of mRNA for the proteasome *α*-subunit C2 (Figure 3C) as well as protein expression of *α*-subunits (Figure 3D and E) increased with increasing weight loss with no evidence for a maximum. As with gastrocnemius muscle there was a linear correlation between pectoral muscle weight and total body weight loss (Figure 3F). Expression of E2 mRNA was little changed with increase in weight loss in pectoral muscle (Figure 4), with a significant increase in expression only being seen in animals with 27% weight loss. This suggests that E2 may not be a rate-limiting step in proteasome proteolysis in this muscle.

## DISCUSSION

Skeletal muscle contains multiple proteolytic pathways for intracellular protein catabolism. Of these, the lysosomal system, including cathepsins B, D and H, is mainly concerned with the digestion of extracellular proteins, although some cytosolic proteins are engulfed in autophagic vacuoles that fuse with lysosomes and are degraded (Dice, 1990). The cytosolic calcium-activated pathway (calpains) appears to play an important role in tissue injury, necrosis and autolysis (Lecker *et al*, 1999). These two processes have been suggested to contribute less than 15–20% towards total protein breakdown in muscle (Attaix *et al*, 1998; Lecker *et al*, 1999) and do not breakdown myofibrillar proteins (Lovell *et al*, 1986). However, the calcium/calpain pathway has been suggested to release myofilaments from the sarcomere in an early and perhaps rate-limiting component of the catabolic response in muscle (Hasselgren and Fischer, 2001). Further catabolism of the actin and myosin released from the myofilaments is considered to occur via the ubiquitin-proteasome proteolytic pathway.

In this process, substrates are marked for degradation through the attachment of a polyubiquitin chain by a series of enzymatic steps mediated by the ubiquitin-activating enzyme (E1), the ubiquitin-conjugating enzyme (E2) and the ubiquitin-protein ligases (E3) (Lecker *et al*, 1999). The polyubiquitinated substrate then enters the proteolytic chamber of the 26S proteasome, where it is unfolded and cleaved to short oligopeptides having mean lengths of 6–9 residues (Kisselev *et al*, 1999). The proteasome is a tube-like structure appearing as a stack of four rings; two outer *α*-rings and two inner *β*-rings in order of *αββα* (Lecker *et al*, 1999). Proteolytic activity is found on the *β*-subunits of the proteasome.

Most studies in cancer patients with weight loss of 10% or higher have suggested that the ubiquitin-proteasome pathway in skeletal muscle shows an increased expression and activity (Williams *et al*, 1999; Bossola *et al*, 2003). However, a study of lung cancer patients referred for curative resection and with a weight loss of only 2.9% showed no increase in expression of components of the ubiquitin-proteasome pathway, while mRNA levels of cathepsin B in skeletal muscle were much higher (Jagoe *et al*, 2002). This suggests that activation of the ubiquitin-proteasome pathway may only occur when weight loss becomes substantial, although Bossola *et al* (2001) reported an increase in ubiquitin mRNA in skeletal muscle of patients when the weight loss was only 5.6±4.9%.

In this study, we have measured the expression of one *α* (C2) and one *β*-subunit (C5) of the proteasome during the progression of loss of gastrocnemius and pectoral muscle in the murine MAC16 cachexia model, as a representative of proteasome structure and function. We chose these genes since protein breakdown in cancer cachexia has been suggested to require increased gene expression of proteasome subunits (Temparis *et al*, 1994) and mRNA levels for proteasome subunits C2 and C5 were found to be increased in extensor digitorum longus (EDL) muscles of rats starved for 2 days, as well as in soleus muscles undergoing denervation atrophy (Medina *et al*, 1995). In addition, we have measured expression of mRNA for the ubiquitin-conjugating enzyme (E2_14k_). Expression of mRNA has been quantitated using RT-competitive PCR, which is based on competitive coamplification of the specific target sequence with an internal standard sharing primer recognition sites in one reaction tube (Auboeuf and Vidal, 1997). Quantitation can then be performed by comparing the PCR signals of the specific template with those obtained with known concentrations of the competitor. Protein expression has also been determined by immunoblotting, since it has been suggested that in various cells elevated concentrations of mRNA of proteasome subunits were not found to be accompanied by increased concentrations of proteasomes (Kanayama *et al*, 1991; Shimbara *et al*, 1992). We have also measured protein expression of E2_14k_.

Using this approach, we have demonstrated a correlation between increases in mRNA and protein expression of proteasome *α* subunits in both gastrocnemius and pectoral muscle. We have also demonstrated a correlation between E2 mRNA and protein levels, although this was less strongly correlated with muscle weight, suggesting that it was not rate-limiting for proteasome proteolysis as might be predicted (Adegoke *et al*, 2002). Proteasome expression and activity in both pectoral and gastrocnemius muscles increased progressively with increasing weight loss up to about 20% followed by a decrease at higher weight loss, except for C2 mRNA in pectoralis muscle, which remained elevated. There was also a decrease in E2 expression at weight losses greater than 20%. Proteasome specific total protein degradation in gastrocnemius muscle, as determined by tyrosine release in the presence of lactacystin, also peaked at 18–20% weight loss and then decreased. A similar rise and fall of the ubiquitin-proteasome pathway was observed in psoas muscle of alloxan-induced diabetic rabbits (Galban *et al*, 2001). Thus, the activity increased 3 and 5 days after diabetes induction, but fell down to control values by day 7, and thereafter decreased below control. In pectoral muscle, both mRNA and protein for proteasome *α*-subunits tended to increase with increasing weight loss, suggesting a lack of coordination between synthesis of *α* and *β* subunits. We have previously shown (Smith and Tisdale, 1993a) that total body nitrogen and the nitrogen content of gastrocnemius muscle decreases with increasing weight loss in mice bearing the MAC16 tumour. The protein content of skeletal muscle is a balance between the rate of protein synthesis and the rate of degradation. Protein synthesis is depressed in muscle of weight-losing mice bearing the MAC16 tumour, and this reduction in protein synthesis may be the major factor contributing to loss of protein at weight losses greater than 20%.

**Figure 5:**
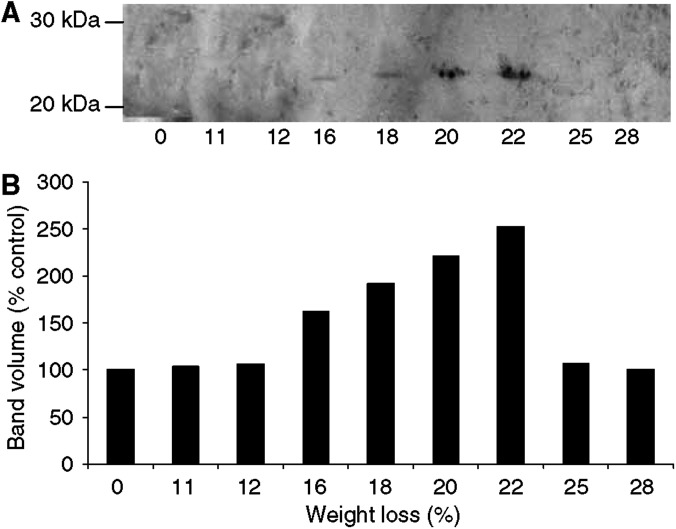
(**A**) Expression of PIF in urinary extracts of mice bearing the MAC16 tumour. Urine (2 ml) was treated with ammonium sulphate (80% w v^−1^) and stirred overnight at 4°C. The precipitated protein was recovered by centrifugation at 3000 ***g*** for 30 min, dialysed against water using an Amicon filtration cell and 15 *μ*g was used for immunoblotting using anti-PIF monoclonal antibody. (**B**) Densitometric analysis of the band intensities in (**A**) reported in arbitary units.

Using the release of tyrosine as a measure of muscle protein degradation breakdown to detect serum factors from the MAC16 tumour that increase protein degradation, activity was found to increase with increasing weight loss up to 20%, and with further weight loss the activity was found to fall to a value not significantly different from that found in animals without weight loss (Smith and Tisdale, 1993b). This circulatory factor has now been isolated and identified (Todorov *et al*, 1996) and shown to be a sulphated glycoprotein of *M*_r_ 24 kDa called proteolysis factor (PIF), which induces an increased expression of both proteasome subunits and E2 in gastrocnemius muscle (Lorite *et al*, 2001). Proteolysis factor has been shown to be responsible for the loss of skeletal muscle in cachectic mice (Lorite *et al*, 1998). Using Western blotting to detect urinary PIF in mice bearing the MAC16 tumour, excretion levels were found to be maximal at weight loss between 20 and 22% (Figure 5). This pattern parallels changes in expression of proteasome subunits in both gastrocnemius and pectoral muscle suggesting that PIF is responsible for the upregulation of the ubiquitin-proteasome pathway. Expression of C2 and C5 mRNA in rectus abdominis muscle of cachectic cancer patients was also found to be maximal at a weight loss of 12–19% (Khal *et al*, 2005). The rate of muscle catabolism, as measured by phenylalanine release, in a rat model of cachexia was also highest at small tumour burdens and decreased as the tumour grew larger (de Blaauv *et al*, 1997). This appeared to be caused by the loss of capacity of the tumour to further break down muscle. The reason for this is not known, but it may be that as the tumour grows and becomes necrotic cells capable of synthesizing PIF are lost. Alternatively, the tumour microenvironment may not be conducive to the synthesis of a highly glycosylated peptide.

The mechanism for the loss of protein in gastrocnemius muscle at weight losses greater than 20% requires further investigation, but the lack of bioactivity of serum from mice bearing the MAC16 tumour at weight losses of 20–25% (Smith and Tisdale, 1993b) suggests that a circulatory factor is not responsible for the protein degradation, and the present results suggest that the ubiquitin-proteasome pathway alone cannot account for the high level of protein breakdown. This suggests that depression of protein synthesis may be more important than an increase in protein degradation at high weight loss.
